# Emergence of a Novel Coronavirus (COVID-19): Protocol for Extending Surveillance Used by the Royal College of General Practitioners Research and Surveillance Centre and Public Health England

**DOI:** 10.2196/18606

**Published:** 2020-04-02

**Authors:** Simon de Lusignan, Jamie Lopez Bernal, Maria Zambon, Oluwafunmi Akinyemi, Gayatri Amirthalingam, Nick Andrews, Ray Borrow, Rachel Byford, André Charlett, Gavin Dabrera, Joanna Ellis, Alex J Elliot, Michael Feher, Filipa Ferreira, Else Krajenbrink, Jonathan Leach, Ezra Linley, Harshana Liyanage, Cecilia Okusi, Mary Ramsay, Gillian Smith, Julian Sherlock, Nicholas Thomas, Manasa Tripathy, John Williams, Gary Howsam, Mark Joy, Richard Hobbs

**Affiliations:** 1 Nuffield Department of Primary Care Health Sciences University of Oxford Oxford United Kingdom; 2 Public Health England London United Kingdom; 3 Vaccine Evaluation Unit Public Health England Manchester United Kingdom; 4 Real-time Syndromic Surveillance Team Public Health England Birmingham United Kingdom; 5 Royal College of General Practitioners London United Kingdom

**Keywords:** general practice, medical record systems, computerized, sentinel surveillance, coronavirus, COVID-19, SARS-CoV-2, surveillance, infections, pandemic, records as topic, serology

## Abstract

**Background:**

The Royal College of General Practitioners (RCGP) Research and Surveillance Centre (RSC) and Public Health England (PHE) have successfully worked together on the surveillance of influenza and other infectious diseases for over 50 years, including three previous pandemics. With the emergence of the international outbreak of the coronavirus infection (COVID-19), a UK national approach to containment has been established to test people suspected of exposure to COVID-19. At the same time and separately, the RCGP RSC’s surveillance has been extended to monitor the temporal and geographical distribution of COVID-19 infection in the community as well as assess the effectiveness of the containment strategy.

**Objectives:**

The aims of this study are to surveil COVID-19 in both asymptomatic populations and ambulatory cases with respiratory infections, ascertain both the rate and pattern of COVID-19 spread, and assess the effectiveness of the containment policy.

**Methods:**

The RCGP RSC, a network of over 500 general practices in England, extract pseudonymized data weekly. This extended surveillance comprises of five components: (1) Recording in medical records of anyone suspected to have or who has been exposed to COVID-19. Computerized medical records suppliers have within a week of request created new codes to support this. (2) Extension of current virological surveillance and testing people with influenza-like illness or lower respiratory tract infections (LRTI)—with the caveat that people suspected to have or who have been exposed to COVID-19 should be referred to the national containment pathway and not seen in primary care. (3) Serology sample collection across all age groups. This will be an extra blood sample taken from people who are attending their general practice for a scheduled blood test. The 100 general practices currently undertaking annual influenza virology surveillance will be involved in the extended virological and serological surveillance. (4) Collecting convalescent serum samples. (5) Data curation. We have the opportunity to escalate the data extraction to twice weekly if needed. Swabs and sera will be analyzed in PHE reference laboratories.

**Results:**

General practice clinical system providers have introduced an emergency new set of clinical codes to support COVID-19 surveillance. Additionally, practices participating in current virology surveillance are now taking samples for COVID-19 surveillance from low-risk patients presenting with LRTIs. Within the first 2 weeks of setup of this surveillance, we have identified 3 cases: 1 through the new coding system, the other 2 through the extended virology sampling.

**Conclusions:**

We have rapidly converted the established national RCGP RSC influenza surveillance system into one that can test the effectiveness of the COVID-19 containment policy. The extended surveillance has already seen the use of new codes with 3 cases reported. Rapid sharing of this protocol should enable scientific critique and shared learning.

**International Registered Report Identifier (IRRID):**

DERR1-10.2196/18606

## Introduction

### Background

The Royal College of General Practitioners (RCGP) Research and Surveillance Centre (RSC) is a network of general practices (GPs) with a nationally representative population [1] that provides pseudonymized data for weekly surveillance of infectious diseases. The disease surveillance program is commissioned by Public Health England (PHE) and covers 37 infectious diseases, including influenza. The RCGP RSC and PHE have an established collaboration of over 50 years in influenza and respiratory disease surveillance [2] and are now in their 53rd season of surveillance and analysis.

The RCGP RSC extracts pseudonymized data from a nationally representative sample of over 500 urban and nonurban GPs each week covering a population of over 4 million. Data from these practices are reported online in a weekly return [3], which includes monitoring weekly rates of influenza-like illness (ILI) and other communicable and respiratory diseases in England. We also produce an annual report [4]. The RCGP RSC data set includes all coded data and all prescribed items including vaccine exposure [1].

**Figure 1:**
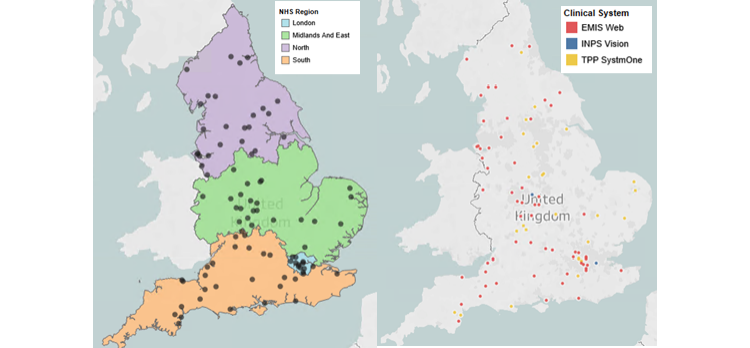
RCGP RSC virology sampling sites. Distribution by National Health Service region and by brand of computerized clinical systems supplier. RCGP RSC has 100 virology sampling sites, there are >500 practices in total signed up to RCGP RSC across England. NHS: National Health Service. RCGP: Royal College of General Practitioners; RSC: Research and Surveillance Centre.

The RCGP RSC conducts virology surveillance each influenza season, with 100 GPs participating in the 2019-2020 season (Figure 1). These virology sampling practices are also recruited to be nationally representative (Figure 1). GPs take nasopharyngeal swabs from persons showing acute respiratory illness within 7 days of the onset of symptoms. Nasopharyngeal swabs are taken from children younger than 5 years showing symptoms of acute bronchitis or bronchiolitis. Additionally, nasopharyngeal samples are taken from anyone 5 years and older showing acute onset of ILI and respiratory synctial virus [5]. Swabs are tested at the PHE Respiratory Virus Unit for influenza to monitor positivity rates and circulating strains, as well as for measuring vaccine effectiveness.

The RCGP RSC successfully conducted a pilot collecting serological samples from adults and linking them to a patient’s medical records during the 2018-2019 influenza season [6]. This pilot was in collaboration with the PHE Seroepidemiology Unit and added to the residual blood samples submitted to PHE by National Health Service (NHS) laboratories [6,7]. Serology can provide important information about background population immunity [6], and sentinel networks can provide a mechanism for systematic data collection and linkage to medical records and health outcomes [8]. The serology pilot has demonstrated the ability of the network to collect serology samples in adults [9].

With the COVID-19 outbreak, PHE and RCGP RSC have adapted existing influenza surveillance to monitor the spread of COVID-19 in the community, and this protocol sets out the basis for that collaboration. The primary national strategy for COVID-19 infection is containment, with patients who are at high risk managed via the telephone help system NHS111 and the PHE health protection teams, but the RCGP RSC surveillance is entirely separate. The RCGP RSC, by extending its established work, will provide virological and serological surveillance to monitor the temporal and geographical distribution of COVID-19 infection in the community, and assess the effectiveness of the containment strategy.

We would not be working in isolation on this research. We will share the protocol with UK colleagues and the I-MOVE consortium who have recently obtained EU Horizon 2020 funding from the stream “Advancing knowledge for the clinical and public health response to the novel coronavirus epidemic” [10]. It is anticipated that great efficiencies in project management will result through this collaboration than that obtained from countries acting alone.

### Aim

The aim of this study is to identify whether there is undetected community transmission of COVID-19, estimate population susceptibility, and monitor the temporal and geographical distribution of COVID-19 infection in the community.

### Objectives

The objectives of this study are as follows:

1. To monitor the burden of suspected COVID-19 activity in the community through primary care surveillance and clinical coding of possible COVID-19 cases referred into the containment pathway
2. To provide virological evidence on the presence and extent of undetected community transmission of COVID-19 and monitor positivity rates among individuals presenting ILI or acute respiratory tract infections to primary care
3. To estimate baseline susceptibility to COVID-19 in the community and estimate both symptomatic and asymptomatic exposure rates in the population through seroprevalence monitoring
4. To pilot implementation of a scheme for collection of convalescent sera with antibody profiles among recovered cases of COVID-19 discharged to the community

We intend to capture the following.

**Figure 2:**
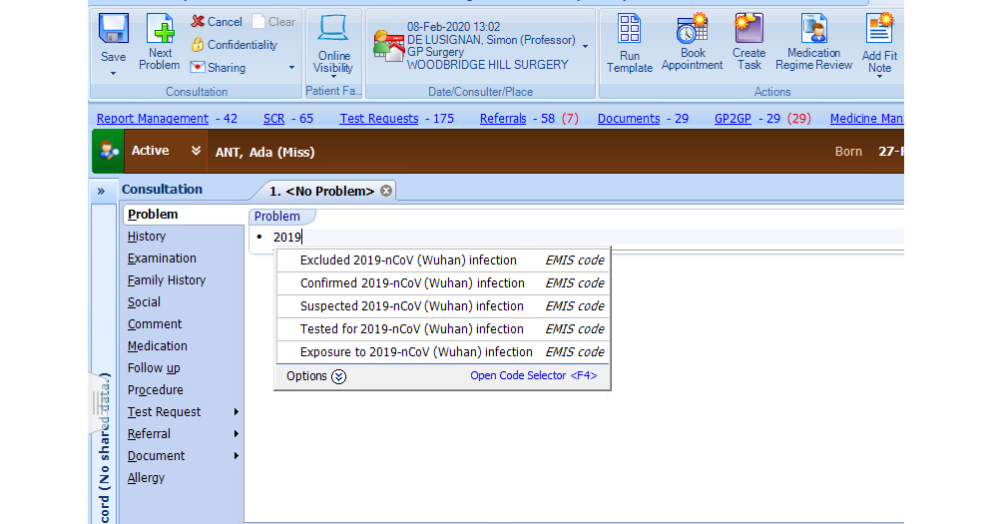
Screenshot of the COVID-19 codes activated in EMIS web. “COVID-19” search terms finds the codes. Ada Ant is NOT a real patient.

**Figure 3:**
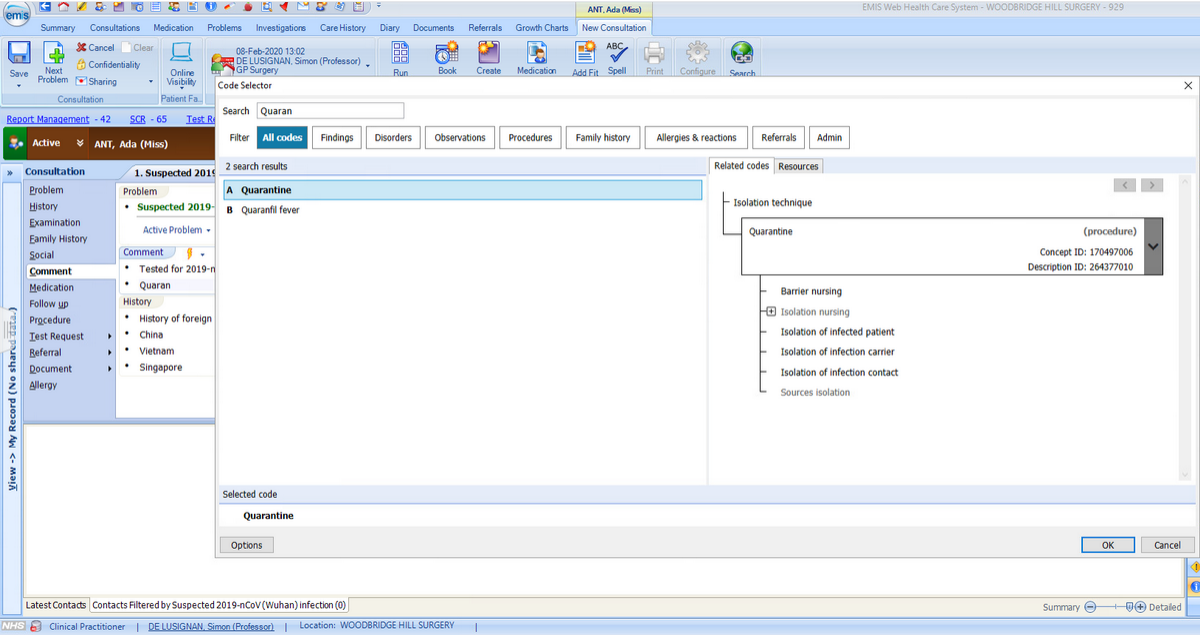
Screenshot showing coding of public health measures in EMIS web.

**Figure 4:**
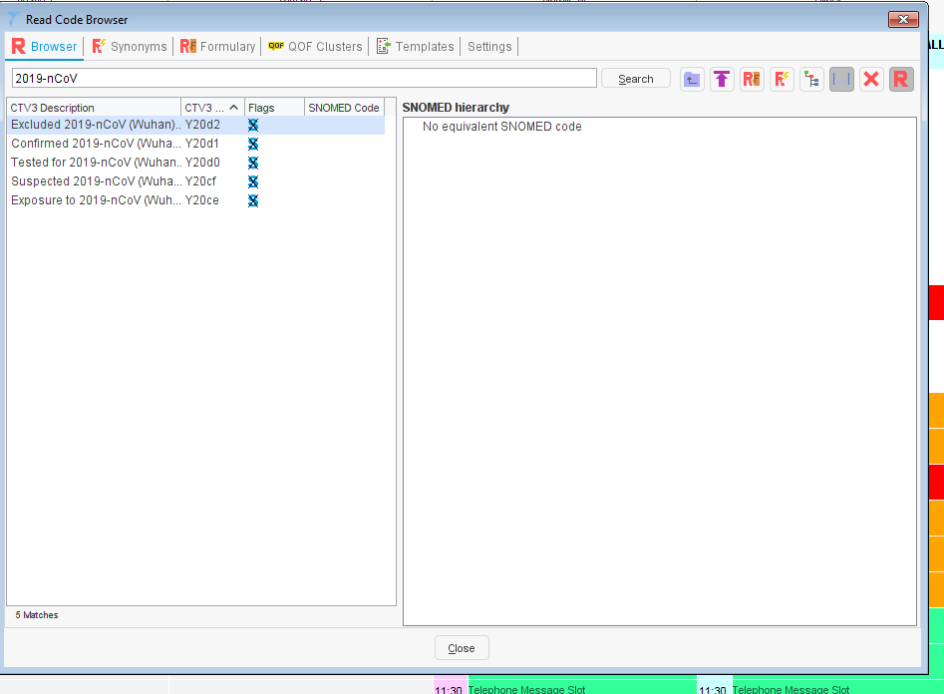
Screenshot of codes available in TPP SystemOne. SNOMED: Systematized Nomenclature of Human Medicine; 2019-nCoV: novel coronavirus.

- Clinical workload related to reports of COVID-19 using the codes created to flag cases, those being assessed and where the infection is located are excluded (Figures 2-4)
- Foreign countries visited in the last 28 days
- Existing codes that may have utility (Tables 1-3). Many GPs and primary care teams may not realize that important relevant data can be coded. There is also the potential during any pandemic to monitor the effectiveness of any transmission control measures.
- Reliable coding of letters and test results that will show an infection has become either confirmed or excluded

**Table 1:** Codes in the Systematized Nomenclature of Human Medicine to flag control measures and audit their effectiveness.

SNOMED^a^ concept ID	Description ID	Preferred term
170497006	264377010	Quarantine
170499009	264381010	Isolation of infection contact
170500000	264384019	Isolation of infection carrier
170503003	264387014	Surveillance of contact
225368008	338663017	Contact tracing
305559001	448017014	Under care of contact tracing nurse
305736005	448259018	Seen by contact tracing nurse
306030003	448793018	Referral by contact tracing nurse
306323004	449303018	Referral to contact tracing nurse
306497009	449538017	Discharge by contact tracing nurse
361235007	477879011	Isolation of infected patient
370835007	1209564019	Monitoring for signs and symptoms of infection
444908001	2871575019	Isolation nursing in negative pressure isolation environment
506931000000109	1126681000000110	Recent travel to disease affected area
710874007	3046686011	Education about cross infection prevention
737612005	3528595017	Education about isolation for infection control
742879000	3550369015	Management of isolation for infection control
9478004	16593015	Prospective focused infection control surveillance

^a^SNOMED: Systematized Nomenclature of Human Medicine.

**Table 2:** Read 2 codes to flag control measures and audit their effectiveness.

Read 2 code	Term
ZV07.00	[V] Need for isolation and other prophylactic measures
65R2.00	Isolation of infection contact
65R3.00	Isolation of infection carrier
65S1.00	Surveillance of contact
65X..00	Contact tracing
8HlA.00	Referral to contact tracing nurse
65R1.00	Isolation of infected patient
13XG.00	Recent travel to disease affected area

**Table 3:** Clinical Terms Version 3 codes to flag control measures and audit their effectiveness.

Clinical Terms Version 3 codes	Preferred terms
ZV07.	[V] Need for isolation and other prophylactic measures
65R2.	Isolation of infection contact
65R3.	Isolation of infection carrier
65S1.	Surveillance of contact
Ua1RW	Contact tracing
XaAQX	Under care of contact tracing nurse
XaATu	Seen by contact tracing nurse
XaAb1	Referral by contact tracing nurse
XaAgt	Referral to contact tracing nurse
XaAk2	Discharge by contact tracing nurse
65R1.	Isolation of infected patient
XaQVi	Recent travel to disease affected area

## Methods

### Overview

The methods will follow the approach used in the current influenza surveillance system [5] and recent serology study [6], and includes five components: (1) primary care clinical surveillance; (2) virological surveillance; (3) population serological surveillance; (4) convalescent sera in cases; and (5) data curation.

### Primary Care Clinical Surveillance

#### Clinical Coding

The NHS uses the Systematized Nomenclature of Medicine Clinical Terms (SNOMED CT) system of coding, which is normally only updated twice annually. There was added complexity as some computerized medical record (CMR) suppliers use the Read coding systems (Read clinical terms version 3 – CTv3), which is no longer updated. Additionally, there were no clinical codes to record COVID-19 in early February 2020. Therefore, the two main GP system suppliers added the five terms shown in Table 4 as system-wide local codes. A UK emergency release of SNOMED CT concepts for COVID-19 was also subsequently made available across all CMR systems (Table 4). The intention is that these will eventually be mapped to the new SNOMED CT concepts as they become available, allowing recording of relevant data (Multimedia Appendix 1).

The key requirements for this release were the ability to code (Table 4) a case of COVID-19, exposure to risk of infection (travel to an area where there may be a higher risk), contact with anyone infected with COVID-19, a report that a person had been tested for COVID-19, and that the disease had been excluded (likely a negative test).

In addition, practices are now able to code any foreign travel undertaken, including the ability to record visits to multiple countries (implemented February 8, 2020). Figures 2-4 show the EMIS web implementation.

Currently, virology samples for influenza surveillance are accompanied by a standard request form.

For COVID-19, we will create a new request form that will record:

- Date of onset of symptoms
- Diagnosis of any of the following:
  Acute bronchitis/bronchiolitis in those younger than five years
  ILI
  Lower respiratory tract infection (LRTI)
- Cough (Y/N)
- History of fever (Y/N); measured (Y/N); if yes, level
- Shortness of breath (Y/N), if measured: oxygen saturation and respiratory rate
- Recent travel (Y/N); if yes, countries visited in last 14 days
- Contact with a named person with confirmed COVID-19 (Y/N) with a free text comment about the level of certainty

These codes will be grouped ontologically into “definite”, “probable”, “possible”, and “not a case” using our standard approach [11] to grouping codes (Table 5), which has been used previously across disease areas [12-14]. The RCGP RSC definition for ILI is shown in Multimedia Appendix 2.

**Table 4:** Local EMIS Health namespace descriptions and TPP system-wide codes for COVID-19.

EMIS Health code description	TPP system-wide code
Excluded 2019-nCoV^a^ (Wuhan) infection	Y20d2
Confirmed 2019-nCoV (Wuhan) infection	Y20d1
Tested for 2019-nCoV (Wuhan) infection	Y20d0
Suspected 2019-nCoV (Wuhan) infection	Y20cf
Exposure to 2019-nCoV (Wuhan) infection	Y20ce

^a^2019-nCov: novel coronavirus.

**Table 5:** Ontological approach to mapping COVID-19 codes.

Category	Code (and its certainty of mapping)	Notes
Confirmed case	Confirmed 2019-nCoV^a^ (Wuhan) infection (Direct mapping codes)	Careful training will be required to ensure validity and reliability. TBC^b^ whether we will require reference lab report
Probable case	N/A^c^	It is possible we will use this category if we do not see data quality problems with definite cases. The WHO^d^ definition is a positive pan-coronavirus assay but without sequencing and absence of other respiratory infections.
Possible case	Exposure to 2019-nCoV (Wuhan) infection Suspected 2019-nCoV (Wuhan) infection Tested for 2019-nCoV (Wuhan) infection (Partially mapping codes)	While awaiting confirmation, we will need to set a time limit (proposed 6 weeks), after which possible cases are reclassified to not a case
Not a case	Excluded 2019-nCoV (Wuhan) infection (Relevant codes with no clear mapping to 2019-nCoV)	As tested patients have negative cases and contacts do not develop symptoms. they will be placed in this category.

^a^2019-nCoV: novel coronavirus.

^b^TBC: to be confirmed.

^c^Not applicable.

^d^WHO: World Health Organization..

#### Public Presentation of Data Using an Observatory and Dashboards

We will develop an observatory to present data nationally and a dashboard for feedback to practices about their data quality and collection of virology and serology samples. This is based on coding described in Table 4.

- Definite case will be presented on our dashboard as “cases” of COVID-19.
- Possible cases will be presented as “Under investigation” (investigating).
- “Not a case” will be presented as “Excluded”.

**Figure 5:**
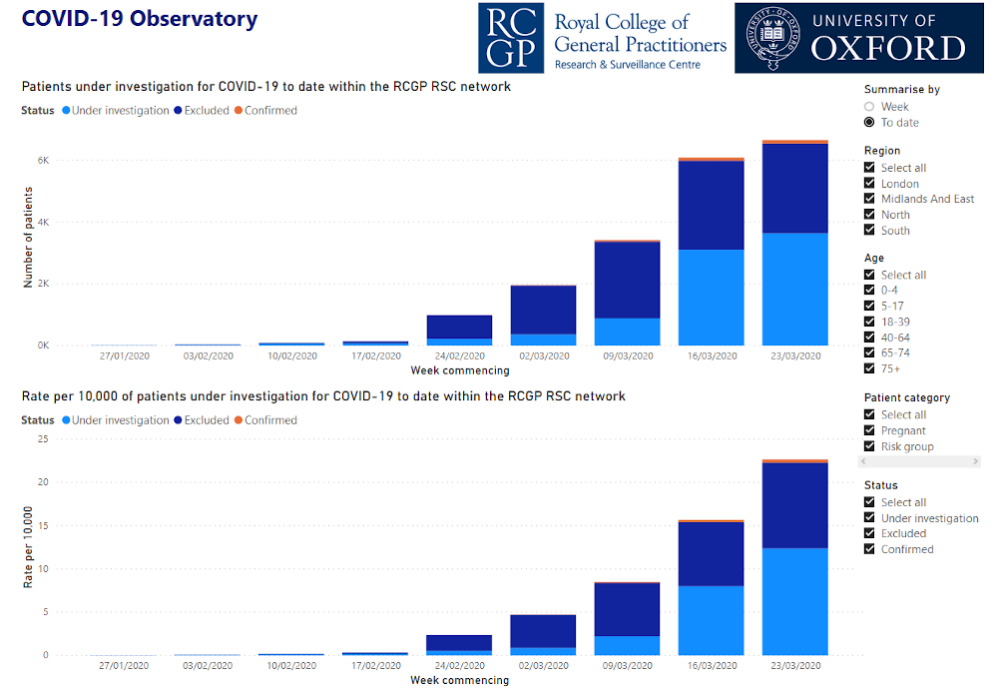
Screenshot of COVID-19 Observatory showing number and rate per 10,000 patients investigated for COVID-19 to date within the RCGP RSC network.

Online data has been established within the initial few weeks in the COVID-19 Observatory (Figure 5), indicating the overall number of patients and rate per 10,000 patients of cases confirmed or under investigation, as well as where the virus is excluded [15].

#### Increasing Report Frequency

We have the option to move to twice weekly surveillance reports with a scope to change this to daily reporting.

### Virological Surveillance

We will continue virology sampling from our sentinel practices, rather than discontinuing as seasonal influenza declines. Additionally, we will recruit more surveillance practices.

The RCGP RSC virology practices will aim to undertake 200-300 nasopharyngeal swabs per week across the RCGP RSC sentinel network, collecting specimens across all age bands. In addition to the inclusion criteria for influenza virology surveillance (ILI, acute bronchitis/bronchiolitis), participating practices will take nasopharyngeal swab samples from any people showing acute symptoms of LRTI if the onset of symptoms is within 7 days.

Sampling will include:

- Taking 4-10 samples per week per practice. RCGP RSC research officers and practice liaison staff will manage practices to achieve a total national sample of 200-300 swabs per week. This could be increased if PHE modelers require more samples.
- Samples from each practice would be spread across the following age groups: <5 years, 5-17 years, 18-64 years, and 65 years and older

Samples (swabs or serum) collected will be sent via prepaid envelopes addressed to the appropriate PHE laboratory for analysis. All samples collected will be tested for the presence of influenza and COVID-19. Additionally, PHE will retrospectively test any influenza virology samples collected between early and mid-February 2020 for COVID-19.

Practices will still follow the PHE protocol [16] for COVID-19 with respect to people at risk of infection who should be signposted down the containment pathway, rather than physically attend their practice. Direct testing of those who attend surgery remains permitted, but we have also rolled out self-swabbing at home [17]. Summary of processes are detailed in Multimedia Appendix 3.

Everyone with an ILI or a respiratory illness who contacts a GP (eg, phones for an appointment) should be asked specifically about recent travel to China and other countries flagged in current PHE advice, or if they have had contact with other people with COVID-19. If these screening enquiries are positive, the patient would be advised to not come to the practice but instead to follow the PHE flow sheet [16]. This can be by a reception or clinician staff, depending on individual practice protocol. These calls should be coded into the GP CMR system and can be reported as part of the RCGP RSC weekly return. We have developed training material to support this coding (Multimedia Appendix 4). These include prompt cards for:

- Practice reception or triage staff: for coding of any patients calling the practice with symptoms of acute respiratory infection with a history of travel to important areas based on PHE advice
- Administrative staff or clinicians who code: to encourage consistent coding of results for any suspected cases, including coding of negative results for exclusion

### Population Serological Surveillance

Practices participating in virology surveillance will opportunistically collect blood samples from patients coming into the practice for a routine blood test. Patients who attend their practice for a routine blood test will be asked to provide an additional sample for serology.

**Figure 6:**
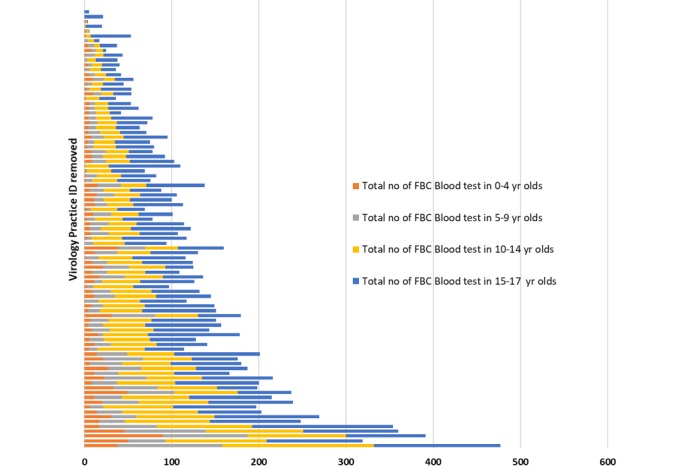
Number of full blood count results in RCGP RSC 2019-2020 virology practices for different child and young adult age groups. Practice unique identifiers have been removed. FBC: full blood count.

**Figure 7:**
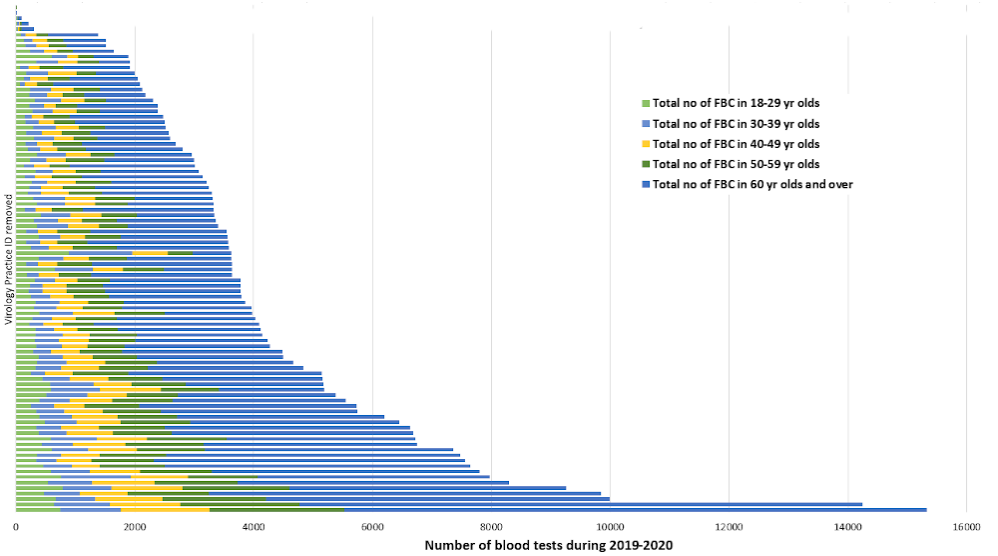
Number of blood tests in Adults.

**Figure 8:**
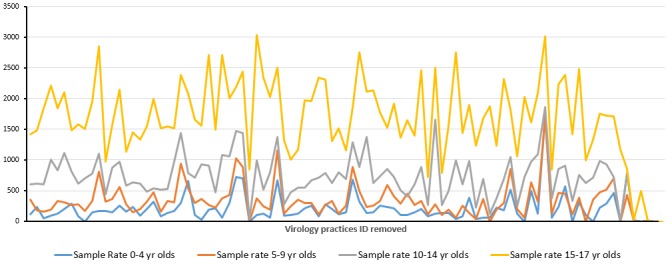
Variation of blood sampling in children and young adults according to age. Data on rate of full blood count sampling per 100,000 registered patients for each children and young adult age groups, per year, by individual virology practice. Practice unique identifiers have been removed.

**Figure 9:**
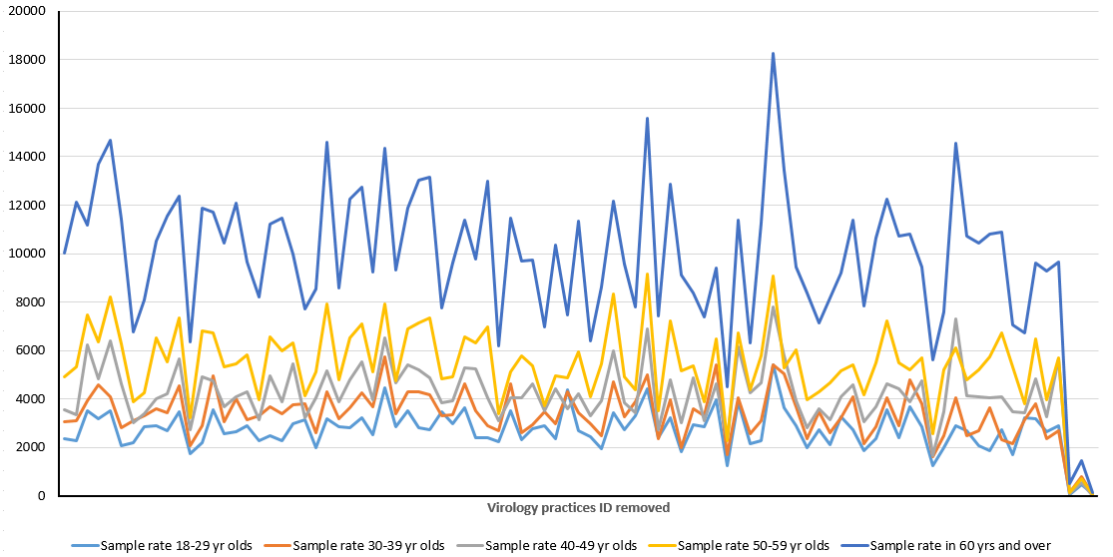
Variation of blood sampling in adults according to age. Rate of full blood count sampling per 100,000 registered patients for each adult age group, per year, by individual virology practice. Practice unique identifiers have been removed.

We have conducted initial searches within the RCGP RSC database to look at the number of full blood count (FBC) results and overall rates in adults and children (Figures 6-9). An FBC is one of the most common tests performed, and we hope this will give an approximate indication of overall numbers of blood tests performed. The sampling rate, per 100,000 patients was highest for children 15-17 years of age and 60 year or older in adults, with the lowest rates in children 0-4 years of age and 18-29 years of age in adults (Figures 6-9).

We will provide 1000 serology baseline samples across all ages that reflect the varying rates of attendance by age. Additionally, we will test if we can obtain these all from virology practices to enhance the yield. A good geographical spread is important, so PHE can advise on areas where serology will most usefully be collected.

This will be followed by 800 samples monthly.

- The sample will be stratified with 200 specimens for prepandemic survey (100 for monthly) in the following age groups: <5 years, 5-17 years, 18-64 years, and 65 years or older.
- The younger patients, in many practices younger than 14 years, and in nearly all for children younger than 8 years will require pediatric serology surveillance.

We will develop a new request form for practices to capture recent travel and exposure to COVID-19.

### Convalescent Sera in Cases

We will pilot a scheme for collecting convalescent serology from people with confirmed cases and who have had an acute virology sample at the time of their infection. This is to identify a carrier state in patients who have recovered from the virus but may continue shedding the virus.

If there are a small number of cases, this may assist in developing a test kit for patients to take to their own GP and explore its acceptability to patients.

If there are a large number of COVID-19 cases nationally, convalescent samples could be collected from RCGP RSC practices where there are confirmed cases, with the ability to link to the full medical record. This process may include checking pseudonymized NHS numbers for positive individuals at RCGP RSC practices, checking current PHE guidance regarding considerations of infectiousness for confirmed cases, and offering the patient an appointment following the previously mentioned process.

This needs to be carefully coordinated nationally across the network and may require PHE to ensure individuals are not contacted by multiple agencies. RCGP RSC could provide a useful structure to channel the initial contact once PHE has made a request. The RCGP RSC practices participating in the annual influenza virology surveillance have started sampling from patients showing symptoms of a LRTI. All samples received are being tested for influenza and COVID-19.

The RCGP RSC will explore ways to collect convalescent samples from any patients tested positive for COVID-19 through the extension of the virological surveillance.

### Data Curation

From the start, we will be carefully curating data to ensure that it can be used for future studies. Our clinical data will be linked to virology. We will curate our data using the Findable, Accessible, Interoperable, Reusable principles. To facilitate this our data set is listed with Health Data Research UK [18] and the European Health Data Evidence Network [19].

### Statistical Methodology

The statistical methodology is in support of a policy approach to widespread disease outbreak, where so-called nonpharmaceutical interventions (NPIs) are used to respond to an emerging pandemic to produce disease suppression. This policy aims to reduce contact rates in the population and thereby reduce transmission of the virus. To implement this the UK government has recently articulated the desire to implement population self-isolation measures. By targeting the reproduction number (R) (the average number of secondary cases each case generates) and aiming to reduce the R to below 1, the policy seeks to reduce case numbers to low levels or (as seen in previous outbreaks with severe acute respiratory syndrome and Ebola) to eliminate human-to-human transmission.

As the experience from the 2009 H1N1 pandemic has shown, NPIs can be a crucial component of pandemic mitigation [20]. Key to the focus of our study will be the estimation of peak cases in the population and continual monitoring by data collection and modelling the potential growth and emergence of subsequent peaks in new cases as social distancing measures are relaxed.

There has already been publication of important disease epidemiological measures concerning the outbreak of COVID-19 in mainland China [21]. A further fundamental measure in pandemic dynamics is the length of time from infection to when a person is infectious to others and the mean duration of infectiousness. These factors, if estimated accurately, will give good predictions for the likely length of the pandemic, the final number of infected cases.

We intend to apply approximate Bayesian inference (ABC) to (possibly spatially heterogeneous) Susceptible-Exposed- Infectious-Removed (SEIR) stochastic epidemic models [22]. Such techniques are highly parallelizable and have been successfully applied to many fields including disease transmission modelling. They are particularly suited to situations where likelihood functions are absent and where more traditional approaches such as Markov chain Monte Carlo are impractical. Such an approach has been demonstrated to work effectively on the ASPREN surveillance data, a network of sentinel GPs and nurse practitioners who report deidentified information on ILIs and other conditions [23], where issues such as missing data and the need to model the observation process itself has been successfully addressed [24]. Furthermore, peaks in new cases have been estimated by distributional methods.

Estimates of the parameters of the SEIR model are tractable on large data sets because of parallelizability, and these methods have been implemented in several R libraries; we intend to use the libraries ABSEIR (deposited on GitHub: https://tinyurl.com/vqu35cj) and abctools (https://tinyurl.com/tfjavz4) to estimate epidemic measures on a weekly basis.

Since we are fitting an SIR-epidemic model in the ABC routine, we anticipate that our results will be robust against weekly case data containing relatively small counts. For example, see [25] for the ABC methodology applied to the Tristan da Cunha common cold data from 1967, where counts of *I* (number of infectious cases) and *R* (number of recovered cases) are in the tens at most.

Finally, in addition to the above methodology we will employ the Kaplan-Meier method with two outcomes (death and recovery) to estimate the case fatality ratio [26]. This approach is independent of the ABC methodology [27] and will allow comparisons between estimates from the two modelling approaches to judge robustness of results.

### Ethical Considerations

RCGP RSC’s surveillance with PHE is defined as *Health Protection* under Regulation 3 of The Health Service (Control of Patient Information) Regulations 2002. This has been confirmed by PHE’s Caldicott Guardian’s Office.

We do not see any increased risk to practices or practitioners taking part in this surveillance. Infection prevention and control advice will follow extant national guidance. Any cases identified will be managed according to the PHE/NHS guidance in force at the time, including advice for identified contacts.

However, our training will include reminders about safe handling of specimens and revision of infection control measures anticipated to be high in our practices. It is a key part of Regulation 12 about safe care and treatment, periodically inspected by the Care Quality Commission [28].

## Results

### Travel History and Clinical Descriptors of the COVID-19 Infections

The RCGP RSC practices have been advised on the clinical coding that has been made available for COVID-19 across all CMR systems. This includes information on coding of clinical descriptors (Table 4) and any recent travel history.

### Establishment of Extended Virology Sampling

The RCGP RSC practices participating in the annual influenza virology surveillance have started sampling from patients showing symptoms of LRTI. All samples received are being tested for influenza and COVID-19. This has led to initial early identification of background spread in low-risk patients.

As of March 7, 2020, the surveillance system has detected 2 cases of COVID-19 in low-risk patients with no history of travel through extended virological sampling.

## Discussion

### Overview

This protocol describes how we have adapted a national influenza surveillance system to monitor community spread of an unexpected infection of COVID-19. We have rapidly created and incorporated new codes to allow data recording, and are collecting data to monitor the effectiveness of containment strategies.

Through this surveillance, we intend to find out more about the epidemiology of COVID-19 in ambulatory care. In particular, its rate of spread, both temporal and geographical. Our testing of low-risk patients will also inform whether the containment strategy that is based on virology testing of high-risk patients and their contacts plus self-isolation is effective. Containment should slow the spread, and there may be benefits in the management of spread from intense surveillance [28]. However, there may come a point at which the virus spreads more widely into the population, as has happened in Italy [29]. Surveillance of low-risk patients should inform when we reach this tipping point and when infection rates start to remit.

The epidemiology of COVID-19 remains emergent [30]. The registration-based nature of UK primary care means that we will be able to create a complete picture of the cumulative incidence and duration.

The surveillance system should be able to identify areas where COVID-19 spread is taking place that might be suitable for trials of antiviral therapy. We could also follow up on the effectiveness or any adverse reactions to these medicines or vaccinations.

Finally, early detection of a confirmed COVID-19 case has exemplified the rapid implementation of this enhanced surveillance in the national network.

### Comparison with Prior Work

Safety of practices is our primary concern. The RCGP RSC has operated for over 50 years and has been involved in collecting samples to monitor disease and vaccine effectiveness through the Hong Kong flu pandemic of 1968/69, the Russian flu of 1977/78, and the 2009 Swine flu pandemic [31,32]. We are not aware of any increased risk to practice staff or other patients from involvement in surveillance. Pandemic preparedness is part of the role of the RCGP RSC.

It is plausible that enhanced coding of information from contacts with the practices in RCGP RSC will reduce the likelihood of people who may be suspected COVID-19 cases being brought to the surgery inadvertently. Where cases are detected unexpectedly, it is probably helpful for that patient, their contacts, and the practice to know. The impact on practices has been to close for a day, if a case is found, for deep cleaning and then reopen.

### Limitations

The principal limitations of our system are the number of data points. We are collecting serology and virology data from 100 sites, which covers a small group of the population. This has been satisfactory for monitoring influenza, but we are not certain if this is a sufficiently large sample for the COVID-19 outbreak. Our sites (surveillance practices) are currently fixed, and it could be helpful to be able to rapidly onboard practices in regions where there are more cases. Currently, we will be reporting weekly. Our existing system can be enhanced to twice weekly, but maybe daily or hourly data should be our current approach.

Opportunistic sampling for serology in children younger than 10 years might be limited due to the overall reduced rate of blood tests in children.

### Conclusions

The extended surveillance using the RCGP RSC-PHE network for the emergent COVID-19 outbreak has been established rapidly. The model of getting the appropriate informatics to enable capture of the required data has already been a success, with data recording starting the week the codes were created. In addition, modifying the existing surveillance system to collect population data in a parallel way has also been effective. However, we are at present unsure as to whether the scale of this surveillance provides sufficient data to drive local containment strategies or if reporting infrequently meets the need of our information age.

## Appendix

Multimedia Appendix 1 New relevant SNOMED CT codes.

Multimedia Appendix 2 The RCGP RSC definition of influenza-like illness.

Multimedia Appendix 3 Main programme.

Multimedia Appendix 4 Training materials for practices.

## Abbreviations

CMR: computerized medical record
FBC: full blood count
GP: general practice
ILI: influenza-like illness
LRTI: lower respiratory tract infection
NHS: National Health Service
NPI: nonpharmaceutical intervention
PHE: Public Health England
R: reproduction number
RCGP: Royal College of General Practitioners
RSC: Research and Surveillance Centre
SEIR: Susceptible-Exposed-Infectious-Removed
SNOMED CT: Systematized Nomenclature of Medicine Clinical Terms
