# Supplementary material for: Emergence of a Novel Coronavirus (COVID-19): Protocol for Extending Surveillance Used by the Royal College of General Practitioners Research and Surveillance Centre and Public Health England
Source: JMIR Public Health Surveill. 2020 Apr 2;6(2):e18606. doi: 10.2196/18606 (PMC7124955; doi:10.2196/18606)
Supplement: Multimedia Appendix 1 [file publichealth_v6i2e18606_app1.docx]

# Appendix 2: New relevant SNOMED CT codes

| UK Edition |
| --- |
| **Clinical finding**  1240581000000104           2019-nCoV (novel coronavirus) detected  1240591000000102           2019-nCoV (novel coronavirus) not detected  1240631000000102           Did not attend 2019-nCoV (novel coronavirus) vaccination  1240751000000100           Disease caused by 2019-nCoV (novel coronavirus)  1240561000000108           Encephalopathy caused by 2019-nCoV (novel coronavirus)  1240571000000101           Gastroenteritis caused by 2019-nCoV (novel coronavirus)  1240601000000108           High priority for 2019-nCoV (novel coronavirus) vaccination  1240531000000103           Myocarditis caused by 2019-nCoV (novel coronavirus)  1240521000000100           Otitis media caused by 2019-nCoV (novel coronavirus)  1240551000000105           Pneumonia caused by 2019-nCoV (novel coronavirus)  1240541000000107           Upper respiratory tract infection caused by 2019-nCoV (novel coronavirus)    **Event**  1240431000000104           Exposure to 2019-nCoV (novel coronavirus) infection    **Observable entity**  1240741000000103           2019-nCoV (novel coronavirus) serology    **Procedure**  1240491000000103           2019-nCoV (novel coronavirus) vaccination  1240511000000106           Detection of 2019-nCoV (novel coronavirus) using polymerase chain reaction technique  1240461000000109           Measurement of 2019-nCoV (novel coronavirus) antibody  1240471000000102           Measurement of 2019-nCoV (novel coronavirus) antigen  1240451000000106           Telephone consultation for suspected 2019-nCoV (novel coronavirus)    **Qualifier value**  1240421000000101           Serotype 2019-nCoV (novel coronavirus)    **Situation with explicit context**  1240661000000107           2019-nCoV (novel coronavirus) vaccination contraindicated  1240651000000109           2019-nCoV (novel coronavirus) vaccination declined  1240781000000106           2019-nCoV (novel coronavirus) vaccination invitation short message service text message sent  1240681000000103           2019-nCoV (novel coronavirus) vaccination not done  1240671000000100           2019-nCoV (novel coronavirus) vaccination not indicated  1240701000000101           2019-nCoV (novel coronavirus) vaccine not available  1240731000000107           Advice given about 2019-nCoV (novel coronavirus) by telephone  1240721000000105           Advice given about 2019-nCoV (novel coronavirus) infection  1240711000000104           Educated about 2019-nCoV (novel coronavirus) infection  1240761000000102           Suspected disease caused by 2019-nCoV (novel coronavirus)    **Substance**  1240401000000105           Antibody to 2019-nCoV (novel coronavirus)  1240391000000107           Antigen of 2019-nCoV (novel coronavirus)  1240411000000107           Ribonucleic acid of 2019-nCoV (novel coronavirus) |
| International edition (emergency update not included in UK release for technical reasons) |
| 840539006 Disease caused by 2019 novel coronavirus (disorder)\|  84053400 \|2019 novel coronavirus vaccination (procedure)\|  840544004 Suspected disease caused by 2019 novel coronavirus (situation)\|  840536004 Antigen of 2019 novel coronavirus (substance)\|  840535000 Antibody to 2019 novel coronavirus (substance)\|  840533007 2019 novel coronavirus (organism)\|  840546002 Exposure to 2019 novel coronavirus (event)\| |

Table 4: The new emergency release of SNOMED CT, UK and International Codes
