# Supplementary material for: Emergence of a Novel Coronavirus (COVID-19): Protocol for Extending Surveillance Used by the Royal College of General Practitioners Research and Surveillance Centre and Public Health England
Source: JMIR Public Health Surveill. 2020 Apr 2;6(2):e18606. doi: 10.2196/18606 (PMC7124955; doi:10.2196/18606)
Supplement: Multimedia Appendix 2 [file publichealth_v6i2e18606_app2.docx]

# Appendix 3: The RCGP RSC definition of influenza-like illness

The RCGP RSC definition of ILI is:

- An acute respiratory illness with a temperature measured/reported/plausibly ≥ 38 °C and cough, with onset within the past 10 days.  ILI cases should not have another more plausible diagnosis. ILI cases have a sudden onset, and there are often symptoms suggestive of systemic upset – myalgia, fatigue, malaise, headache etc.  RCGP RSC stresses the notion of within 10 days of onset to differentiate acute episodes, with swabs only wanted within 7 days of onset.
- This definition is compatible with the World Health Organisation (WHO) and European Centre for Disease Control (ECDC) definitions.
- The WHO definition has the highest specificity (21.4%) and ECDC the highest sensitivity (96.1%)
