# Supplementary material for: Emergence of a Novel Coronavirus (COVID-19): Protocol for Extending Surveillance Used by the Royal College of General Practitioners Research and Surveillance Centre and Public Health England
Source: JMIR Public Health Surveill. 2020 Apr 2;6(2):e18606. doi: 10.2196/18606 (PMC7124955; doi:10.2196/18606)
Supplement: Multimedia Appendix 3 [file publichealth_v6i2e18606_app3.docx]

# Appendix 5: Main programme

#

## 1. Initiation

We propose a phased implementation with the following first steps taking place in parallel to the detailed programme of work:

- 1. RCGP RSC Virology sampling practices to continue and expand collection of virology swabs from people with ILI and lower respiratory tract infections.
  2. RCGP RSC to start using the COVID-19 codes now available

## 2. Main programme

Serology and other activities to be initiated.

We will commence four activities, the first three during the initiation phase, with the serology component starting later.

### Activity 1: Collection of additional data by reception / triage team:

Primary care team members answering the phone, conducting triage, or responding to online enquiries about ILI or ARI should ask if there has been any foreign travel.If the answer is yes, they should follow PHE’s advice about suspected COVID-19. They should code into the clinical record as set out below

Coding advice:

1. Code the respiratory illness, as well as can be diagnosed over the phone
2. Code *Suspected COVID-19 / Suspected* 2019-nCoV where there has been foreign travel or other reason that person is suspected of infection.
3. Or if there has been a contact with a person with COVID-19 then the *Exposure to COVID-19 / Exposure to 2019-nCoV* code should be used. N.B. 2019-nCoV is the same as COVID-19 and these codes may need searching for using
4. Where there is foreign travel within 28 days please code: code *history of foreign travel,* and also code the countries visited in the last 28 days
5. If a confirmed case, or someone in quarantine/self-quarantine who is getting in touch with the surgery please code relevant data (Table 3).

### Activity 2: Ensuring cases reported to the practices and tests are coded

All the clinicians and staff who code in the practice should code confirmed cases (where there is strong evidence – e.g. discharge summary, or virology result). All the cases should be coded. Where a history is unclear then use the suspected or exposed codes.

Where there is a negative test result, then the *COVID-19 Excluded/* 2019-nCoV *Excluded* code should be used.

Activity 3: Collecting virology swabs from people with ILI or lower respiratory infections
We will be collecting virology swabs, as now for surveillance from people who present to the surgery. THESE ARE LOW RISK PEOPLE WHO DO NOT FALL INTO THE PHE SUSPECTS COVID-19 CATEGORY ABOVE. People with ILI or LRTIs should be swabbed. We are looking for COVID-19 spreading in the ambulatory population. Swabs and virology material are in place already for this to take place.

### Activity 4: Collecting an extra blood bottle for serology from well people attending for routine blood tests

We will also be collecting blood samples, an extra bottle from people who are attending for blood samples anyway.

## 3. Detailed data collections – if required

Additional data collections will take place as agreed.

## 4. Data curation and return to standard surveillance

The final three months will be spent curating data to ensure effective evaluation of a many interventions/public health measures as possible.
