# Supplementary figures and images for: Emergence of a Novel Coronavirus (COVID-19): Protocol for Extending Surveillance Used by the Royal College of General Practitioners Research and Surveillance Centre and Public Health England
Source: JMIR Public Health Surveill. 2020 Apr 2;6(2):e18606. doi: 10.2196/18606 (PMC7124955; doi:10.2196/18606)

# Appendix 6: Training materials for practices

**
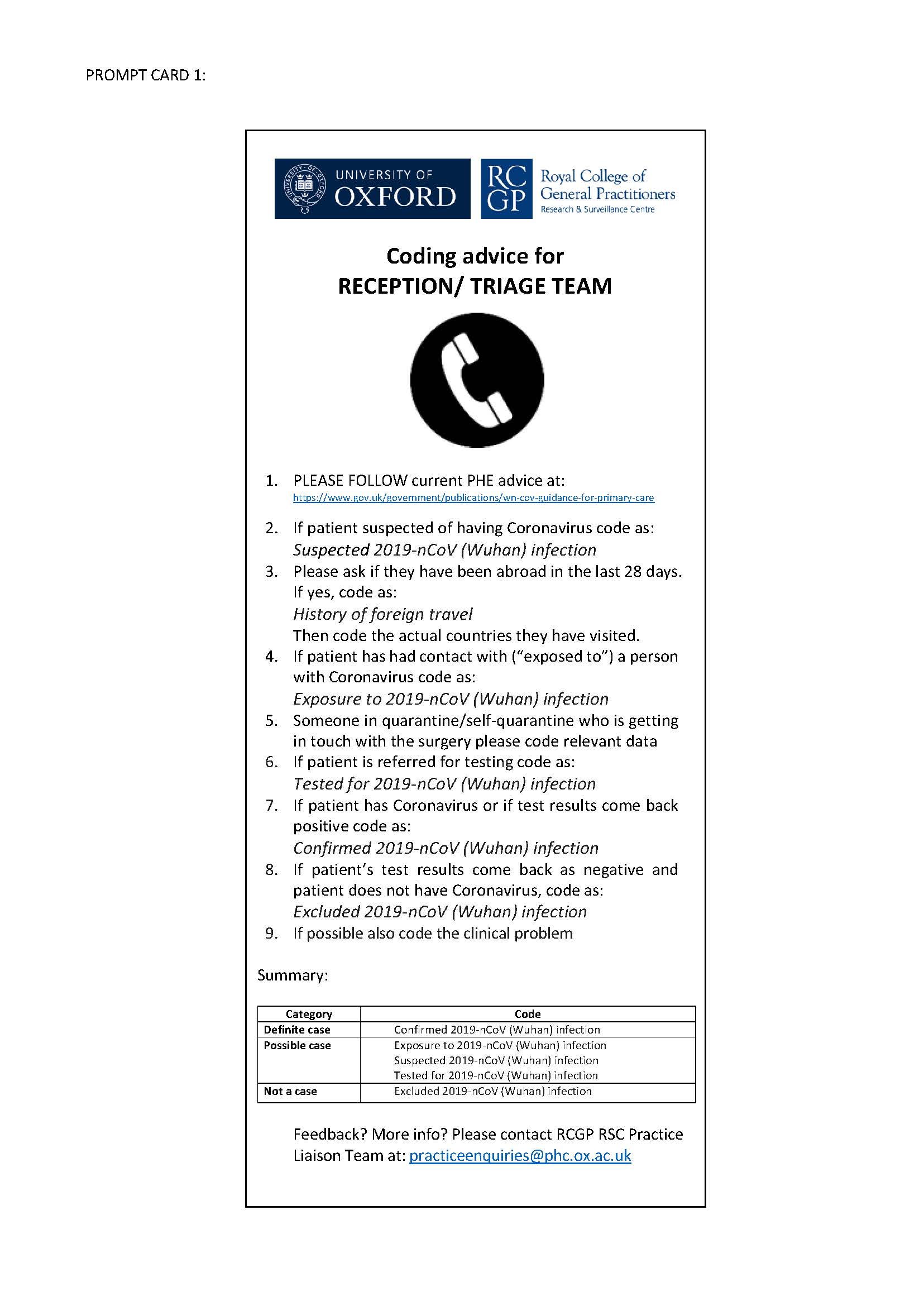
**


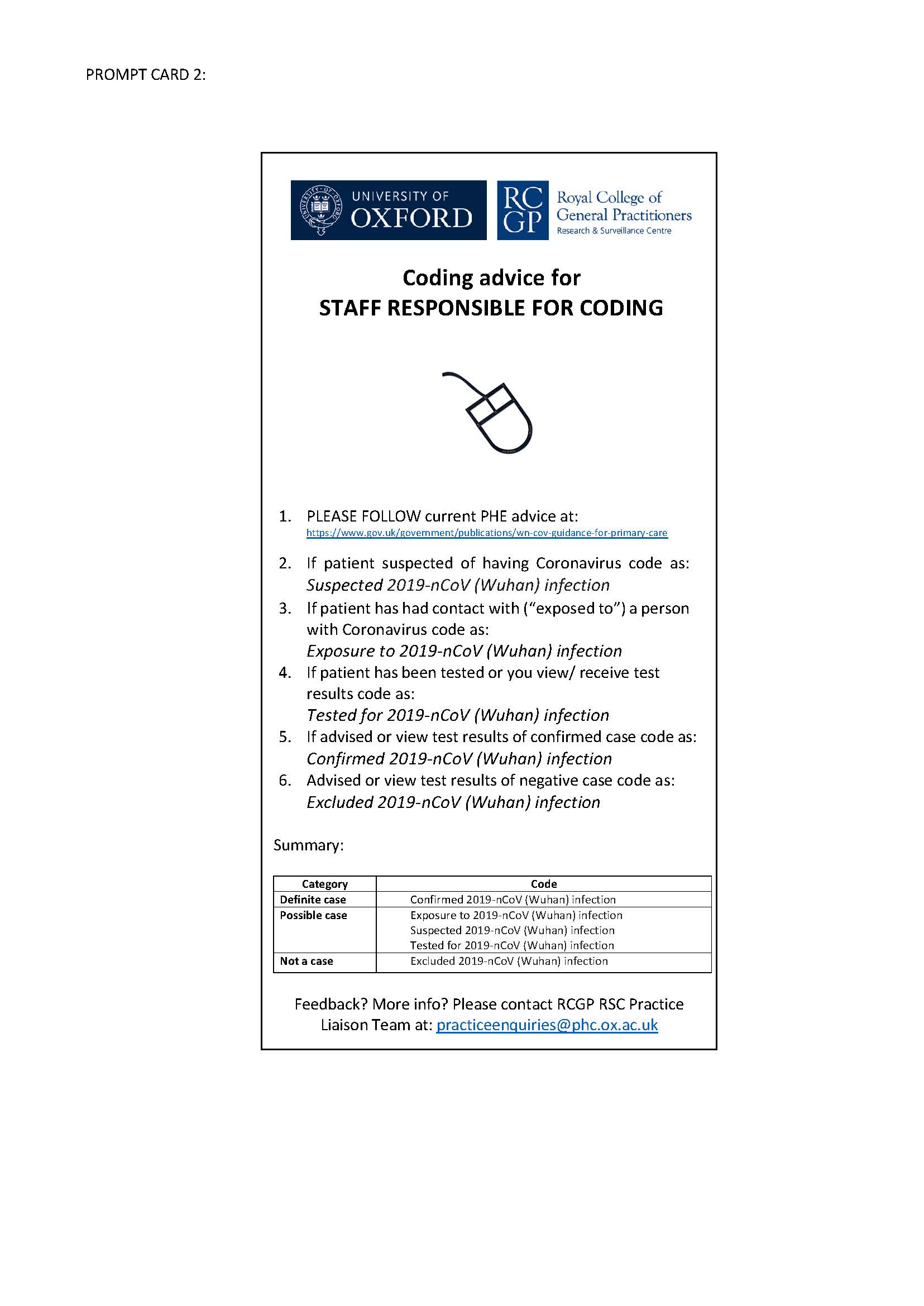


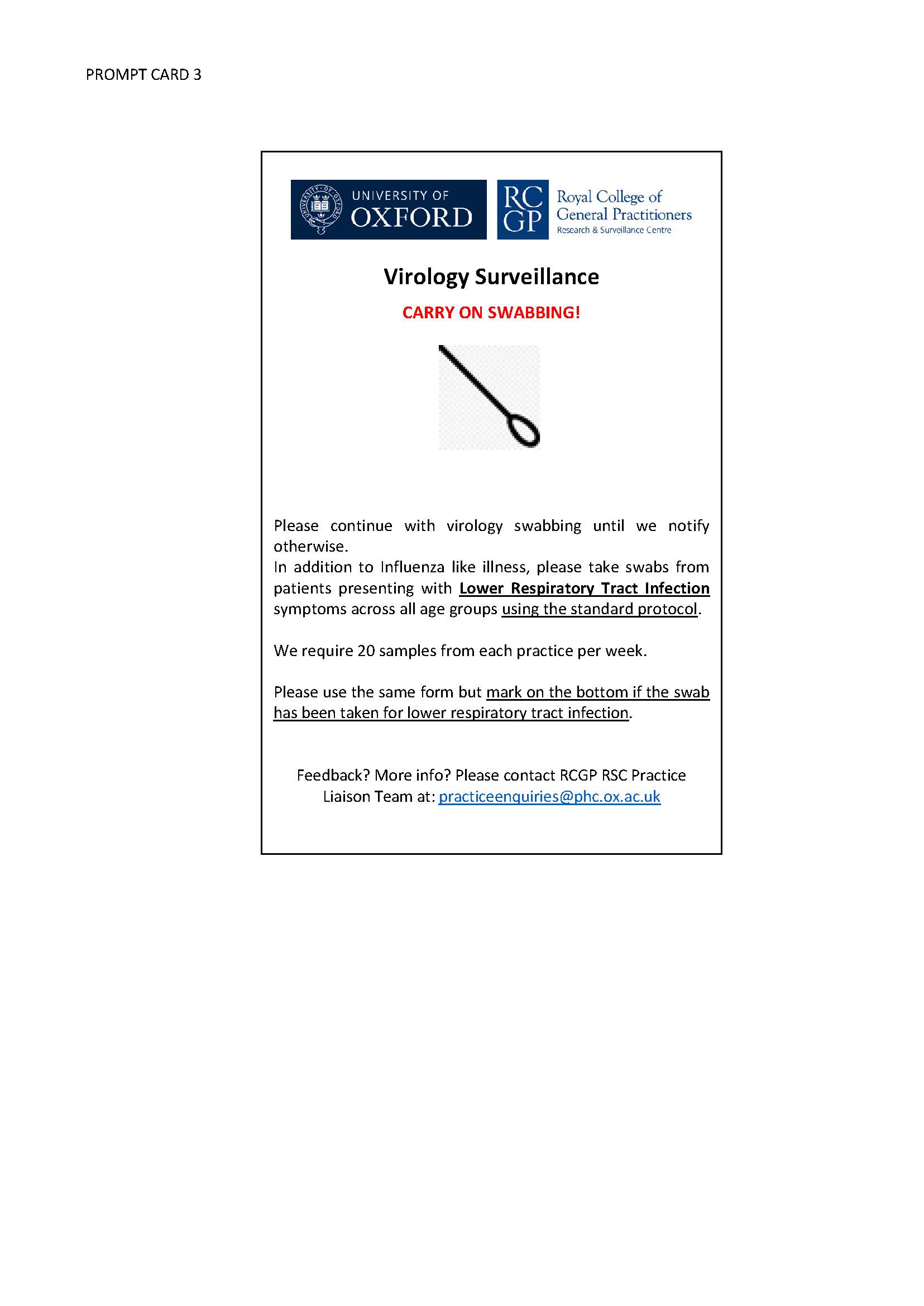

Supplement: Multimedia Appendix 4 [file publichealth_v6i2e18606_app4.docx]
